# Green tea and the risk of breast cancer: pooled analysis of two prospective studies in Japan

**DOI:** 10.1038/sj.bjc.6601652

**Published:** 2004-02-24

**Authors:** Y Suzuki, Y Tsubono, N Nakaya, Y Suzuki, Y Koizumi, I Tsuji

**Affiliations:** 1 Division of Epidemiology, Department of Public Health and Forensic Medicine, Tohoku University Graduate School of Medicine, Sendai 980-8575, Japan

**Keywords:** green tea, breast cancer, prospective cohort study

## Abstract

In a pooled analysis of two prospective studies with 35 004 Japanese women, green-tea intake was not associated with a lower risk of breast cancer (222 cases), the multivariate relative risk for women drinking ⩾5 cups compared with <1 cup per day being 0.84 (95% confidence interval 0.57–1.24, Trend *P*=0.69).

Although laboratory studies have suggested a protective effect of green tea on breast cancer risk, few epidemiological studies have examined the association. A case–control study of Asian Americans in the United States found a lower risk of breast cancer in association with green-tea intake (Wu *et al*, 2003), whereas a prospective cohort study in Japan found no association (Key *et al*, 1999). To further examine the association between green-tea consumption and the risk of breast cancer, we conducted a pooled analysis of two population-based prospective cohort studies among women in rural northern Japan.

## MATERIALS AND METHODS

Details of the design of the two cohort studies are described elsewhere (Tsubono *et al*, 2001; Nakaya *et al*, 2003). Briefly, Cohort 1 started in 1984 and included 17 353 women aged ⩾40 years (94% response rate) (Tsubono *et al*, 2001); Cohort 2 started in 1990 and included 24 769 women aged 40–64 years (93% response rate) (Nakaya *et al*, 2003). Self-administered questionnaires covered recent (Cohort 1) or usual (Cohort 2) consumption of green tea and used the same five frequency categories ranging from ‘never’ to ‘⩾5 cups per day’. It had a reasonably high level of validity and reproducibility (Ogawa *et al*, 2003). After exclusion of those with missing responses or with a prior history of cancer, 14 409 subjects in Cohort 1 and 20 595 in Cohort 2 remained. We followed up vital and residential status of the study subjects by population registries. Through population-based cancer registries, 103 incident cases of breast cancer were identified in Cohort 1 (9 years follow-up with 111 267 person-years) and 119 in Cohort 2 (7 years follow-up with 151 882 person-years).

We estimated relative risk (RR) and 95% confidence interval (CI) of breast cancer according to green-tea consumption, using Cox's proportional-hazards regression with the adjustment for age and potential confounders. To obtain a summary measure of results between Cohorts 1 and 2, the general variance-based method was used to pool each RR and 95% CI. We repeated all analysis after excluding breast cancer cases diagnosed in the first 3 years of follow-up (38 in Cohort 1 and 40 in Cohort 2). *P*-values for the test of linear trend were calculated by treating the green-tea consumption category as an ordinal variable. All reported *P*-values are two-tailed.

## RESULTS

Table 1

**Table 1:** Characteristics of the subjects according to green-tea consumption

	**Green-tea consumption (cups/day)**			
	**Cohort 1**		**Cohort 2**	
**Characteristics**	**<1**	**⩾5**	**<1**	**⩾5**
No.	2614	6185	5879	5409
Age (years), means±s.d.	57.4±12.1	58.2±10.7	50.7±7.5	54.2±7.0
				
*Types of health insurance* (%)				
National Health Insurance	57.3	56.7	49.3	56.5
Others	42.7	43.2	50.7	43.5
				
*Age at menarche (years)* (%)				
⩽13	19.3	17.4	28.1	20.9
14	21.6	20.0	24.6	24.1
15	23.4	24.7	22.1	23.9
⩾16	35.7	37.9	25.2	31.1
				
*Menopausal status* (%)				
Premenopause	37.4	30.3	50.8	32.2
Postmenopause	62.6	69.7	49.2	67.8
				
*Parity* (%)				
Nulliparous	0.3	0.3	2.4	2.8
1–2	43.9	41.7	49.4	47.5
⩾3	55.8	58.0	48.2	49.7
				
*Age at first birth among the parous (years)* (%)				
⩽21	19.8	21.6	17.6	16.8
22–23	25.2	26.0	26.7	28.5
24–25	25.8	25.2	30.4	28.8
⩾26	29.2	27.2	25.4	25.9
				
*Mother's history of breast cancer* (%)				
Presence	0.6	0.6	0.6	0.7
Absence	99.4	99.4	99.4	99.3
				
*Smoking* (%)				
Never	88.5	85.1	87.9	88.1
Past	3.5	3.3	2.8	1.8
Current	8.0	11.6	9.4	10.2
				
*Alcohol drinking* (%)				
Never	67.3	62.1	69.3	71.5
Past	5.0	3.7	4.2	3.8
Current	27.7	34.3	26.5	24.8
				
*Body mass index* (%)				
<18.5	6.9	5.6	3.0	2.5
18.5–24.9	66.7	64.3	68.3	61.8
⩾25.0	26.3	30.1	28.7	35.7
				
*Consumption frequencies of black tea (cups/day)* (%)				
Never	58.5	45.9	64.6	59.3
Occasionally	35.5	45.7	33.6	37.2
1–2	4.9	6.3	1.6	2.3
⩾3	1.2	2.1	0.3	1.2
				
*Consumption frequencies of coffee (cups/day)* (%)				
Never	35.1	26.2	20.8	24.3
Occasionally	34.6	47.6	32.2	45.5
1–2	23.4	19.8	34.3	23.0
⩾3	6.9	6.5	12.8	7.2

compares the characteristics of subjects in the highest and the lowest categories of green-tea consumption. Subjects in Cohort 1 with higher intake tended to be postmenopausal, while such women in Cohort 2 tended to be slightly older, postmenopausal and have a higher body mass index. Subjects in both cohorts with a lower intake tended to drink black tea less frequently and coffee more frequently.

We found no inverse association between green-tea intake and the risk of breast cancer, whether data for Cohorts 1 and 2 were combined or separated (Table 2

**Table 2:** RR of breast cancer according to green-tea consumption

	**Green-tea consumption (cups/day)**				
**Variable**	**<1**	**1 or 2**	**3 or 4**	**⩾5**	**Trend *P*-value**
*No. of cases of breast cancer/person-years*					
Cohort 1	17/19 607	10/18 200	26/24 795	50/48 665	
Cohort 2	37/43 213	30/35 415	30/33 264	22/39 990	
					
*Age-adjusted RR*					
Cohort 1	1.00	0.63 (0.29–1.38)	1.21 (0.66–2.24)	1.20 (0.69–2.07)	0.21
Cohort 2	1.00	0.99 (0.61–1.60)	0.06 (0.65–1.71)	0.65 (0.38–1.10)	0.17
Pooled	1.00	0.87 (0.60–1.28)	1.11 (0.76–1.63)	0.87 (0.59–1.28)	0.86
					
*Multivariate PR1*					
Cohort 1	1.00	0.66 (0.30–1.44)	1.20 (0.65–2.22)	1.17 (0.67–2.05)	0.26
Cohort 2	1.00	0.96 (0.59–1.57)	1.00 (0.61–1.63)	0.61 (0.36–1.06)	0.12
Pooled	1.00	0.87 (0.57–1.32)	1.07 (0.73–1.57)	0.84 (0.57–1.24)	0.69
					
*Multivariate PR2*					
Cohort 1	1.00	0.33 (0.11–1.03)	0.95 (0.45–1.99)	0.96 (0.50–1.86)	0.51
Cohort 2	1.00	1.04 (0.54–1.99)	1.64 (0.90–2.96)	0.85 (0.43–1.66)	0.95
Pooled	1.00	0.78 (0.44–1.37)	1.32 (0.83–2.10)	0.90 (0.56–1.45)	0.63

a Multivariate relative risk (RR) has been adjusted for age, types of health insurance, age at menarche (⩽13, 14, 15 and ⩾16 years), menopausal status (premenopause and postmenopause), age at first birth (⩽21, 22–23, 24–25 and ⩾26 years), parity (nulliparous, 1–2 and >3), mother's history of breast cancer, smoking (never, past and current smoking), alcohol drinking (never, past and current drinking), body mass index (<18.5, 18.5–24.9 and ⩾25.0) and consumption frequencies of black tea and coffee (never, occasionally, 1–2 and >3 cups per day).

RR1 denotes the relative risk with all cases of breast cancer included in the multivariate analysis, and RR2 the relative risk with cases diagnosed in the first 3 years of follow-up excluded from the analysis. Values in parentheses are 95% confidence intervals.

). Exclusion of cases of breast cancer diagnosed in the first 3 years of follow-up did not substantially change the results and nor did further adjustment for consumption frequencies of vegetables, fruits and meat.

When we performed stratified analyses according to variables used as potential confounders in the multivariate analysis, the association between the consumption of green tea and breast cancer risk was not substantially modified. In a stratified analysis according to soybean soup intake (less than daily and daily), the pooled multivariate RRs (95% CI) of breast cancer for women who drank five or more cups per day, as compared with women who drank less than one cup per day, were 0.95 (0.29–3.10; Trend *P*=1.00) among those consuming soybean soup less than daily (35 cases) and 0.81 (0.54–1.24; Trend *P*=0.63) among those consuming soybean soup daily (185 cases).

We found no relation between breast cancer risk and the consumption of black tea and coffee. The pooled multivariate RRs (95% CI) compared with women who never drank black tea were 0.90 (0.64–1.27) for those drinking black tea occasionally and 1.44 (0.77–2.69) for those drinking one or more cups per day (Trend *P*=0.81). The corresponding risks for coffee were 0.78 (0.53–1.13) and 0.81 (0.55–1.18) (Trend *P*=0.44).

## DISCUSSION

We found no association between green-tea consumption and breast cancer incidence among Japanese women who consumed green tea much more frequently than women in Western countries. The results agree with a prospective study (Key *et al*, 1999) but disagree with a case–control study (Wu *et al*, 2003). In the latter paper, the authors suggested that breast cancer risk might be influenced by the intake of both green tea and soy, and that the benefit of green tea was primarily observed among subjects who were low soy consumers. We examined this possibility but found no differential association between green-tea consumption and the risk of breast cancer among high and low consumers of soybean soup.

Two hospital-based cohort studies (Nakachi *et al*, 1998; Inoue *et al*, 2001) observed a nonsignificant lower risk of recurrence among high green-tea consumers than among low green-tea consumers with breast cancer, although such an effect may not apply to healthy women.

We also found no relationship between the risk of breast cancer and the consumption of black tea or coffee. Our results are consistent with the judgment of World Cancer Research Fund that it is possible that black tea has no relationship with breast cancer risk and that it is convincing that coffee has no relationship with the risk of breast cancer (World Cancer Research Fund, 1997).

In conclusion, in a pooled analysis of two prospective cohort studies in rural northern Japan, we found no association between consumption of green tea, coffee or black tea and the risk of breast cancer.
